# An FYVE-Domain-Containing Protein, PsFP1, Is Involved in Vegetative Growth, Oxidative Stress Response and Virulence of *Phytophthora sojae*

**DOI:** 10.3390/ijms22126601

**Published:** 2021-06-20

**Authors:** Jinhui Zhang, Xiaoran Du, Xin Zhou, Duo Jin, Jianqiang Miao, Xili Liu

**Affiliations:** 1 State Key Laboratory of Crop Stress Biology for Arid Areas, College of Plant Protection, Northwest A&F University, 3 Taicheng Road, Yangling 712100, China; jh.zhang@nwafu.edu.cn (J.Z.); duxiaoran@nwafu.edu.cn (X.D.); xinchou@nwsuaf.edu.cn (X.Z.); jinduo@nwafu.edu.cn (D.J.); 2 Department of Plant Pathology, College of Plant Protection, China Agricultural University, 2 Yuanmingyuanxi Road, Beijing 100193, China

**Keywords:** FYVE, PX, *Phytophthora sojae*, CRISPR/Cas9, gene knockout, pathogenicity

## Abstract

Proteins that contain the FYVE zinc-finger domain are recruited to PtdIns3P-containing membranes, participating in numerous biological processes such as membrane trafficking, cytoskeletal regulation, and receptor signaling. However, the genome-wide distribution, evolution, and biological functions of FYVE-containing proteins are rarely reported for oomycetes. By genome mining of *Phytophthora sojae*, two proteins (PsFP1 and PsFP2) with a combination of the FYVE domain and the PX domain (a major phosphoinositide binding module) were found. To clarify the functions of PsFP1 and PsFP2, the CRISPR/Cas9-mediated gene replacement system was used to knock out the two genes respectively. Only heterozygous deletion mutants of *PsFP1* were recovered, and the expression level of *PsFP1* in the heterozygous knockout transformants was significantly down-regulated. These *PsFP1* mutants showed a decrease in mycelial growth and pathogenicity and were more sensitive to hydrogen peroxide. These phenotypes were recovered to the level of wild-type by overexpression *PsFP1* gene in the PsFP1 heterozygous knockout transformant. In contrast, deletion of *PsFP2* had no significant effect on vegetative growth, asexual and sexual reproduction, pathogenicity, or oxidative stress sensitivity. PsFP1 was primarily localized in vesicle-like structures and both the FYVE and PX domains are important for its localization. Overall, our results indicate that *PsFP1* plays an important role in the vegetative growth and virulence of *P. sojae*.

## 1. Introduction

The FYVE domain is a zinc-finger binding domain that notably occurs in fungi, metazoans, and oomycetes. [1,2,3]. The FYVE acronym was derived from the names of the first four proteins recognized to contain this domain: Fab1p, YOTB, Vac1p, and EEA1 (early endosomal antigen-1) [4]. FYVE domains are approximately 60–70 amino acids in length and contain eight conserved cysteine residues. Furthermore, these domains contain three conserved motifs: an N-terminal WxxD motif followed by a highly characteristic R(R/K)HHCR motif and a C-terminal RCV motif [1,2,5]. These motifs, especially the R(R/K)HHCR motif, determine its interaction with phosphatidylinositol 3-phosphate (PI3P) [6,7]. The interaction between the FYVE domain and PI3P was found to be very specific, and almost no other phosphoinositides can bind to FYVE [8].

PI3P is one of the products of phosphatidylinositol 3′-kinase (PI3K). Unlike other products of PI3K, PI3P can be detected in normal cells and mainly distributed on early endosomes and vesicles of multivesicular endosomes [6]. The results of many studies have shown that PI3P plays an important role in regulating cell base and necessary physiological activities such as signaling cascades and intracellular membrane trafficking [9,10]. Furthermore, PI3P is involved in the infection process of several oomycete and fungal plant pathogens. Previous studies have shown that the effector proteins AvrLm6 from *Leptosphaeria maculans* and Avr1b from *Phytophthora sojae* can enter plant cell cytoplasm by binding PI3P on the extracellular surface of plant cells [11,12]. Recent work reported that *P. sojae* can generate PI3P and that PI3P was enriched in haustoria during the infection stage [13]. Given the high affinity and specificity of the interaction between the FYVE domain and PI3P, FYVE domain-containing proteins may exert multiple functions by binding PI3P in many different contexts.

*P. sojae* is an economically relevant plant pathogen, which can cause root and stem rot of soybean and bring huge economic loss to soybean production every year. [14]. With the completion and publication of the *P. sojae* genome sequence, it has gradually become a model species for the study of oomycete plant pathogens [15]. A total of 272 proteins containing the FYVE domain were found in *P. sojae* by genome mining. This is a much higher number than those in humans (27 proteins), yeast (5 proteins), and *Arabidopsis thaliana* (15 proteins) [1,16,17]. In eukaryotes, the FYVE domain is usually combined with other domains. Previous studies showed that 63% of FYVE proteins contain at least one other domain, and 58 domains have been found to combine with FYVE [18]. Among these domains, five (Ank, WD40, Beach, PH, and PIP5K) are found to combine with the FYVE domain in almost all taxonomic groups, while the distribution of the other domains may vary in different species. However, there are few reports about the function of proteins containing the FYVE domain in plant pathogens.

Like the FYVE domain, the PX domain is a major phosphoinositide-binding module. Previous studies have shown that several PX domains can specifically bind some phosphatidylinositols: not only PI3P but also PI4P, PI(3,4)P_2_, PI(4,5)P_2_, and PI(3,4,5)P_3_ [19,20,21,22,23,24,25]. In eukaryotes, there are few studies on proteins containing both FYVE and PX domains; indeed, only one such protein has been reported: the protein kinase ZFK in *Trypanosoma brucei* [26,27]. Deletion of ZFK resulted in reduced growth of bloodstream-form parasites in culture. Genome mining of *P. sojae* revealed that two proteins (Ps349585 and Ps481659) containED both an FYVE and a PX domain. They were named PsFP1 and PsFP2, respectively. Until now, there are no reports about the function of FYVE domain-containing proteins in oomycete. Thus, in this study, we would like to clarify the role of two FYVE domain-containing proteins, PsFP1 and PsFP2, in *P*. *sojae* using the CRISPR/Cas9 system.

## 2. Results

### 2.1. Sequence Analysis of PsFP1 and PsFP2

**Figure 1:**
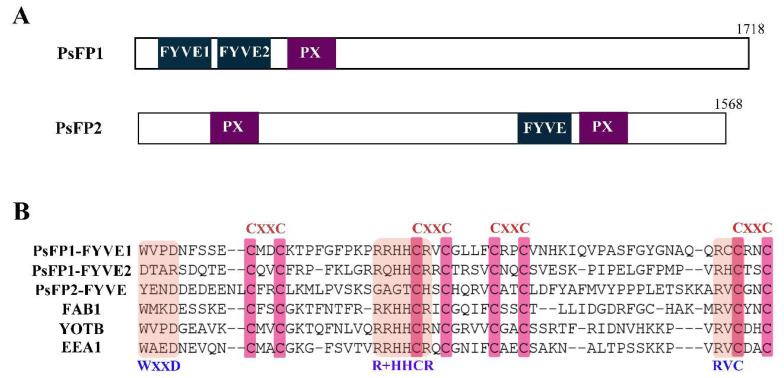
Characteristics of PsFP1 and PsFP2. (**A**) Detailed domain structures of PsFP1 and PsFP2. PsFP1 contains two FYVE domains at the N-terminus, followed by a PX domain. PsFP2 contains a PXA domain at the N-terminus and an FYVE and a PX domain at the C- terminus; (**B**) Alignment of FYVE domains present in PsFP1, PsFP2, and other classical FYVE-containing proteins (FAB1, YOTB, and EEA1). The position of eight zinc-coordinating cysteine residues and three conserved PI3P binding motifs (signature WxxD, R + HHCR, and RVC) are marked in the figure. (+) stands for lysine or arginine.

Analysis of the domain organization of the 272 FYVE-containing proteins in *P. sojae* revealed that only two proteins, PsFP1 (protein ID: 349585) and PsFP2 (protein ID: 481659), harbored the FYVE-PX bigram. PsFP1 and PsFP2 encode proteins of 1718 and 1568 amino acids, respectively (Figure 1A). PsFP1 contains two FYVE domains at the N-terminus, followed by a PX domain. PsFP2 contains a PXA domain (a domain associated with the PX domain) at the N-terminus with an FYVE and a PX domain located at the C-terminus.

By aligning the FYVE domains of PsFP1, PsFP2, and other classical FYVE containing proteins (FAB1, YOTB, and EEA1), it was found that the FYVE domains of PsFP1 are highly conserved, but the FYVE domain of PsFP2 showed only limited sequence identity (Figure 1B). The FYVE1 domain of PsFP1 has the canonical features of an FYVE domain, including eight conserved cysteine residues, the PI3P-binding site with the consensus sequence R + HHCR, and the WxxD motif in the N-terminus. However, the RVC motif in the C-terminus is missing in the FYVE1 domain of PsFP1. Compared with the FYVE1 domain, the FYVE2 domain of PsFP1 has a Q instead of an R in the PI3P-binding motif and is missing the WxxD motif. In contrast, the FYVE domain of PsFP2 has only the eight conserved cysteine residues and the RVC motif.

### 2.2. Expression Analysis of PsFP1 and PsFP2

**Figure 2:**
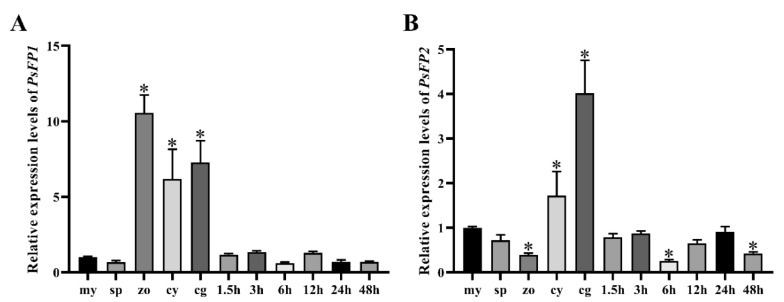
Expression patterns of *PsFP1* and *PsFP2* at different stages. Relative expression levels of *PsFP1* (**A**) and *PsFP2* (**B**) at developmental and post-infection stages were measured by qRT-PCR. In this figure, my represents mycelia, sp represents sporulating hyphae, zo represents zoospore, cy represents cysts and cg represents cyst germination, while 1.5 h, 3 h, 6 h, 12 h, 24 h, and 48 h represent different periods of the infection stage. A one-way ANOVA was used for statistical analysis, and asterisks indicate significant differences (*p* < 0.01).

*PsFP1* and *PsFP2* were expressed in all stages described above. The *PsFP1* expression level was significantly up-regulated (more than six-fold) in zoospores, cysts, and germinating cysts, compared to within mycelia (Figure 2A). In addition, the *PsFP2* expression level was up-regulated in cysts and germinating cysts compared with mycelia (by approximately two- and four-fold, respectively) (Figure 2B). During infection, the *PsFP1* and *PsFP2* expression levels were the same as or lower than in the mycelium stage. This result suggested that *PsFP1* and *PsFP2* may play an important role during development, especially in the zoospore, cyst, and germinating cyst stages.

### 2.3. Generation of PsFP1 or PsFP2 Deletion Transformants

**Figure 3:**
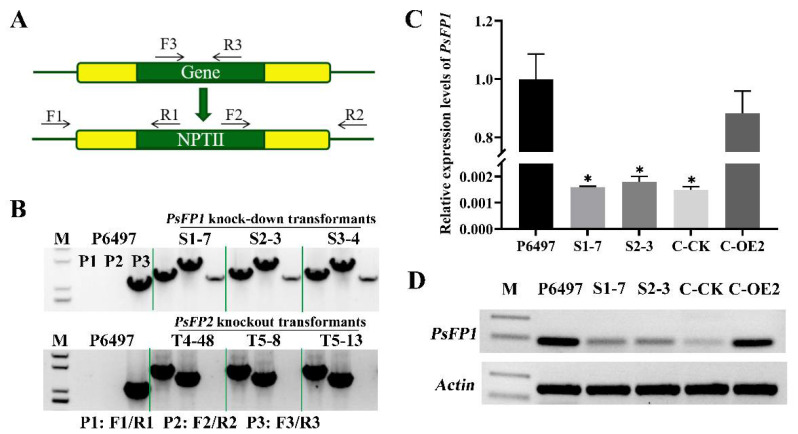
The verification of *PsFP1* and *PsFP2* transformants. (**A**) Primer locations for PCR verification are marked using black arrows; (**B**) PCR analysis of three representative mutants of *PsFP1* or *PsFP2* knockout transformants, demonstrating that the PsFP1 knockout transformants (S1-7, S2-3, S3-4) are heterozygotes, whereas the PsFP2 knockout transformants (T4-48, T5-8, T5-13) are homozygotes. The relative expression levels of *PsFP1* in *PsFP1* knock-down transformants, complemented transformant (C-OE2) and empty control line of *PsFP1* knock-down transformant (C-CK) were measured using (**C**) qRT-PCR and (**D**) semi-qRT-PCR. M: molecular marker. A one-way ANOVA was used for statistical analysis, and asterisks indicate significant differences (*p* < 0.01).

For *PsFP1*, no homozygous deletion transformants were obtained despite checking over 800 transformants from at least six independent transformation experiments. Only six heterozygous knockout transformants of *PsFP1* (S1-7, S1-10, S2-3, S2-5-S6, S2-5-S51, S3-4) were obtained, and three independent transgenic lines are shown in Figure 3B. For *PsFP2*, we obtained six homozygous deletion mutants (T4-48, T4-49, T5-8, T5-9, T5-13, T5-14), and three independent transgenic lines are shown in Figure 3B. We measured the *PsFP1* expression level in the heterozygous knockout transformants and found that it was markedly down-regulated (Figure 3C). Semi-qRT-PCR results also showed that the expression of *PsFP1* was significantly decreased in the heterozygous knockout transformants (Figure 3D). Therefore, these transformants can be used as *PsFP1* knock-down transformants to test the functions of *PsFP1* in *P. sojae*.

### 2.4. The Phenotypes of P. sojae Transformants

**Figure 4:**
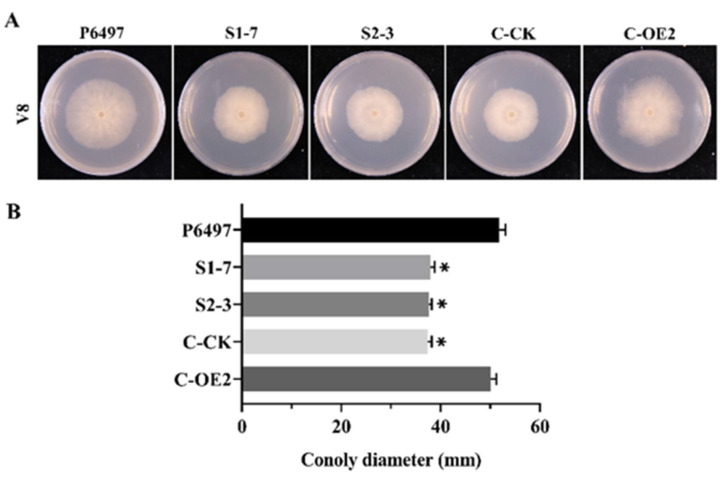
Mycelia growth rate of the *PsFP1* knock-down mutants. (**A**) Mycelia growth of the *PsFP1* knock-down mutants on V8 medium. The wild type strain P6497, *PsFP1* knock-down transformants (S1-7, S2-3), complemented transformant (C-OE2) and empty control line of *PsFP1* knock-down transformant (C-CK) were inoculated on V8 medium and cultured at 25 °C for 5 days; (**B**) Statistical analysis of mycelia growth rate on V8 medium. A one-way ANOVA was used for statistical analysis, and asterisks indicate significant differences (*p* < 0.01).

The growth rate of *PsFP1* knock-down transformants decreased by approximately 30% compared with that of the wild-type P6497 (Figure 4 and Table S1). In contrast, the growth rate of *PsFP2* knockout transformants was almost the same as that of P6497 (Table S2). Furthermore, *PsFP1* knock-down transformants and *PsFP2* knockout transformants exhibited similar phenotypes to that of P6497 in sporangial production, zoospore release, cyst germination, and oospore production assays. To further confirm the decline of mycelial growth rate is related to *PsFP1*, we carried out gene complementation by overexpression of *PsFP1* in *PsFP1* knock-down transformants. And the *PsFP1* knock-down transformant with the empty vector served as control. Then, the qPCR was used to detect *PsFP1* expression in the transformants to determine whether the *PsFP1* was successful complementation. The results showed that the *PsFP1* expression in complemented strain (C-OE2) was restored, which was basically consistent with that of wild strain P6497 (Figure 3C,D). Meanwhile, the expression level of *PsFP1* in the control strain (C-CK) was similar to the *PsFP1* knock-down transformants. Then, we found that the mycelial growth rate of the complemented strain C-OE2 was basically the same as that of the wild type P6497, while the growth rate of the control strain C-CK was the same as that of the *PsFP1* knock-down transformants (Figure 4). Therefore, *PsFP1*, rather than *PsFP2*, is involved in mycelial growth, and loss of neither *PsFP1* nor *PsFP2* affects the asexual and sexual reproduction of *P. sojae*.

### 2.5. PsFP1 Is Required for Full Virulence of P. sojae

**Figure 5:**
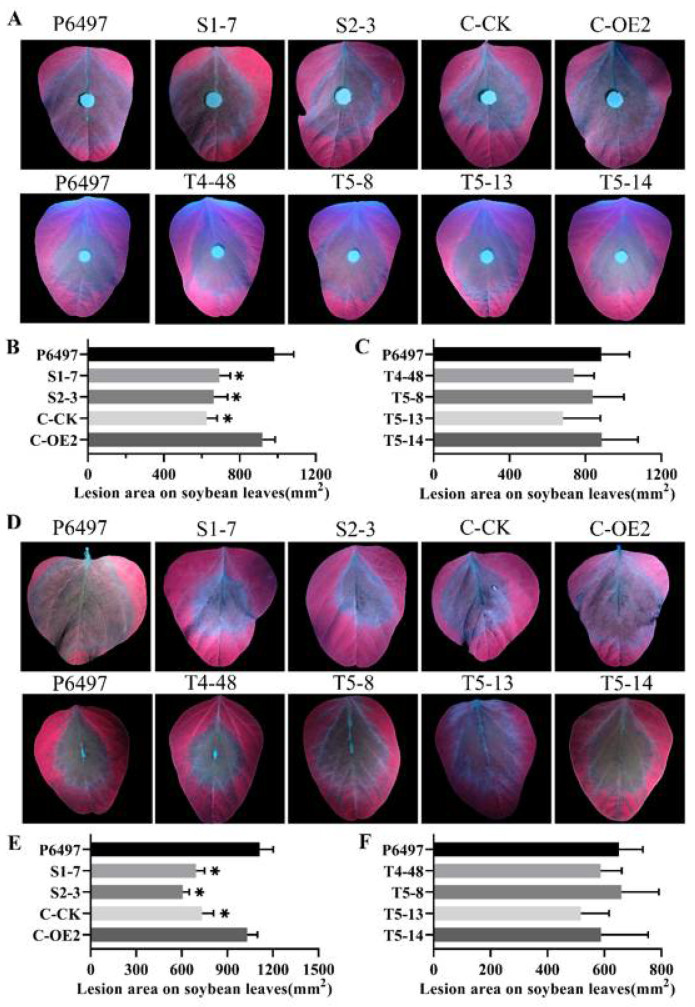
Virulence of *PsFP1* knock-down transformants (S1-7, S2-3), *PsFP1* complemented transformant (C-OE2), empty control line of *PsFP1* knock-down transformant (C-CK), and *PsFP2* knockout transformants (T4-48, T5-8, T5-13, T5-14). Virulence phenotypes were determined by inoculating soybean leaves (Williams soybean cultivar) with mycelial plugs or ~200 zoospores. After inoculation with mycelia, (**A**) disease symptoms were photographed and (**B**,**C**) lesion areas were measured to evaluate the severity of disease at 2 days post-inoculation (dpi). After inoculation with zoospores, (**D**) disease symptoms were photographed and (**E**,**F**) lesion areas were measured at 3 dpi. A one-way ANOVA was used for statistical analysis, and asterisks indicate significant differences (*p* < 0.01).

The lesion area of *PsFP1* knock-down transformants was significantly smaller than that of P6497 and the complemented transformant C-OE2 (Figure 5A,B). However, the lesion area of *PsFP2* knockout transformants was similar to that of P6497 (Figure 5A,C). We also tested the pathogenicity of zoospores of these strains on soybean leaves, and similar results were obtained (Figure 5D–F). These results indicate that *PsFP1* is essential for the pathogenicity of *P. sojae*.

### 2.6. PsFP1 Is Important for P. sojae to Manage with Oxidative Stress

**Figure 6:**
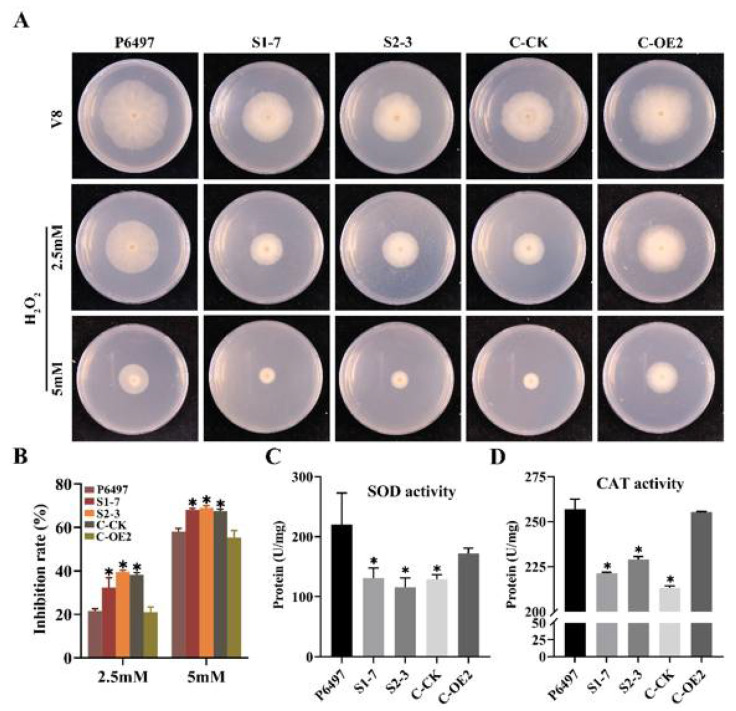
The phenotype of the P6497 and *PsFP1* mutants under different oxidative stress. (**A**) Mycelia growth of the P6497, the *PsFP1* knock-down mutants (S1-7, S2-3), *PsFP1* complemented transformants (C-OE2), and empty control line of *PsFP1* knock-down transformant (C-CK) under oxidative stress. These strains were inoculated on V8 medium with or without 2.5 or 5 mM H_2_O_2_ and cultured at 25 °C for 7 days; (**B**) Statistical analysis of mycelia growth inhibition rate with or without H_2_O_2_; (**C**) The SOD enzyme activity in *PsFP1* knock-down transformants; (**D**) The CAT enzyme activity in *PsFP1* knock-down transformants. A one-way ANOVA was used for statistical analysis, and asterisks indicate significant differences (*p* < 0.01).

Under different concentrations of H_2_O_2_, the mycelial growth inhibition rate of *PsFP1* knock-down transformants was significantly lower than that of the P6497 and the complemented transformant C-OE2 (Figure 6A,B), indicating that *PsFP1* knock-down transformants were more sensitive to H_2_O_2_. We also analyzed the superoxide dismutase (SOD, the key enzyme for ROS production) and catalase (CAT, the key enzyme for ROS degradation) activity, the results showed that the SOD and CAT activity were significantly decreased in *PsFP1* knock-down transformants (Figure 6C,D). Therefore, we speculate that *PsFP1* may be involved in the signaling pathway mediating resistance to oxidative stress in *P. sojae*. Additionally, the temperature sensitivity test showed that there was no significant difference between the *PsFP1* knock-down transformants and P6497 at different temperatures (Figure S1).

### 2.7. Subcellular Localization of PsFP1 is Determined by the FYVE and PX Domains

**Figure 7:**
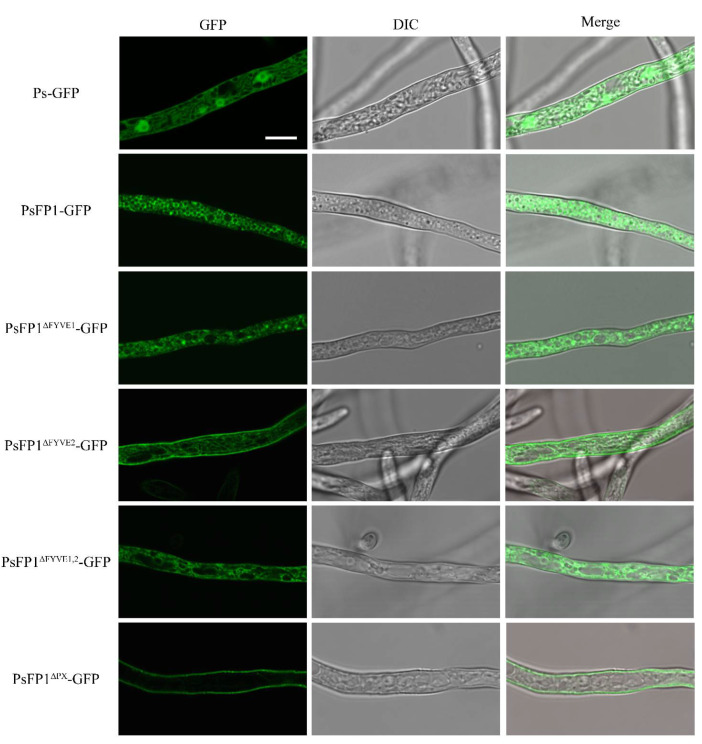
Subcellular localization of PsFP1 and truncation mutants lacking FYVE or PX domain in vegetative mycelia of *P. sojae*. The PsFP1-GFP fusion protein shows vesicle-like localization, while the PsFP1^ΔFYVE1^-GFP, PsFP1^ΔFYVE2^-GFP, and PsFP1^ΔFYVE1,2^-GFP are weakened in vesicle-like positioning. The PsFP1^ΔPX^-GFP losses the vesicle-like localization and instead appeared to be localized at the plasma membrane. Bars = 10 μm.

In the PsFP1-GFP transformants, the GFP fluorescence was primarily localized to vesicle-like structures, which were numerous (Figure 7). The deletion of the FYVE or PX domain weakened the GFP fluorescence in vesicle-like structures; indeed, in the PX domain deletion transformants, the GFP signal at vesicle-like structures disappeared. These results indicate that both the FYVE and PX domains of PsFP1 are necessary for proper subcellular localization.

## 3. Discussion

In eukaryotes, the FYVE domain is conserved in many proteins. Previous studies have shown that the FYVE domain can specifically bind to PI3P and that FYVE domain-containing proteins participate in membrane trafficking, signal transduction, cytoskeleton regulation, and other biological processes [28,29,30]. A total of 272 FYVE proteins can be found in *P. sojae*, a much higher number than reported for other eukaryotes [1,17,31]. Moreover, the transcription pattern and domain composition of these proteins are clearly different, which suggested that these proteins might play different important functions in *P. sojae*.

In this study, the only two proteins in *P. sojae* that contain both the FYVE and PX domain architecture were identified. In recent years, the CRISPR/Cas9 gene knockout system has been successfully applied in *P. sojae*, providing an important method for the study of gene function in Phytophthora [32,33]. The efficiency of the CRISPR/Cas9 knockout system has always been one of the main common concerns in the user community. The genomic background of target DNA, the percentage of GC, chromatin structure, and the secondary structure of sgRNA are the main factors affecting cleavage efficiency [34,35,36,37]. In this study, we failed to obtain homozygous deletion mutants for *PsFP1*. The GC percentage of *PsFP1* is 65%, which should not affect the efficiency of *PsFP1* cleavage, and three different sgRNAs with good secondary structures were designed for the *PsFP1* gene knockout. Therefore, we speculate that the chromatin structure of *PsFP1* may have affected the cleavage efficiency in the CRISPR/Cas9 system. Although no homozygous transformants were obtained, heterozygous transformants can also be to study the function of *PsFP1*, because the *PsFP1* gene is hardly expressed in heterozygous transformants. Previous studies have shown that allelic expression imbalance exists widely in eukaryotes [38,39,40]. Thus, the significant decrease in *PsFP1* expression in heterozygous transformants may be due to the deletion of the preponderant allele of *PsFP1*.

Endocytosis and exocytosis are essential for membrane trafficking-dependent growth of many plant pathogens [41,42,43]. In *Fusarium graminearum*, Vps27 is a component of ESCRT-0 (endosomal sorting complexes required for transport-0) and is involved in the multivesicular body (MVB) sorting pathway during endocytosis, and the deletion of Vps27 caused a reduction in growth rate [41]. Furthermore, the FYVE domain of Vps27 can bind PI3P at the membrane of endosomes, and this binding is vitally important for the localization and function of Vps27 in the MVB sorting pathway [5,44]. Our observation indicated that PsFP1 is located on the membrane of a vesicle-like structure, which indicates that knock-down of *PsFP1* may affect the membrane trafficking process and, further, may affect vegetative growth. This is similar to the phenotype observed upon altering the expression of the FYVE protein Fab1 in *A. thaliana* [26,27]. Fab1a and Fab1b were located in the endosomes of root epidermal cells. An imbalance of expression of Fab1a and Fab1b destroyed the homeostasis of endomembrane systems, affecting endocytosis, vacuolar formation, and vacuolar acidification, and resulting in most pleiotropic developmental phenotypes involving auxin signaling transduction [45].

The pathogenicity of pathogens in plants is regulated by many factors. Pathogen growth states, including mycelial growth rate, spore germination, and formation of infection structure, have a substantial influence on their pathogenicity [41,46,47]. In the current study, the mycelial growth rate of *PsFP1* knockdown transformants was inhibited, which may be one of the reasons why pathogenicity was affected for these transformants. In addition, during the process of infection, pathogens can secrete distinct pathogenic factors to combat plants’ immune responses [48,49,50,51]. Previous studies have shown that PI3P can mediate the entry of RXLR effectors into plant cells [11], and the FYVE domain can specifically bind to PI3P, so we speculate that an FYVE protein may also participate in this process. Moreover, plants have evolved many effective defense systems against pathogens, among which reactive oxygen species (ROS) such as H_2_O_2_ are considered one of the fastest [52]. Meanwhile, the pathogens also have a corresponding inhibition or degradation mechanism to inhibit the host’s defense response, such as catalase (CAT), which plays a very important role in pathogen resistance and detoxification of H_2_O_2_ [53,54]. In this study, the significant decrease of CAT activity in *PsFP1* knockdown transformants may lead to the decrease of their ability to degrade H_2_O_2_ produced by plants, thus affecting their pathogenicity. Furthermore, pathogens can also produce ROS, and the disruption of intracellular ROS homeostasis will lead to functional defects. In the endophytic fungus *Epichloe festucae*, ROS plays a negative regulatory role, preventing the excessive proliferation of fungi, so that fungi and host plants can survive together [55]. Knockout of NADPH oxidase, the key enzyme for ROS production in *Magnaporthe oryzae* and *Botrytis cinerea*, resulted in decreased pathogenicity [56,57]. In the *PsFP1* knockdown transformants, the SOD and CAT activity were significantly decreased, which may lead imbalance of intracellular ROS level, and then may affect the mycelial growth and pathogenicity. So we suspect that the *PsFP1* is involved in important mechanisms for balancing intracellular and extracellular ROS levels in. According to this hypothesis, the knockdown of *PsFP1* resulted in ROS imbalance, which led to a decrease in mycelial growth and pathogenicity.

In general, FYVE domain-containing proteins are poorly studied in plant pathogenic oomycetes. In our study, only two proteins (PsFP1 and PsFP2) containing both FYVE and PX domains were found in. The biological functions of PsFP1 and PsFP2 on growth, development, responses to different types of stress, and virulence were further studied in with a CRISPR/Cas9-mediated knockout. Future research will focus on the function of other FYVE proteins in, and precisely dissect the hypothetic interacting proteins and the potential regulatory pathways responsible for the corresponding phenotype. Our results will enrich our understanding of the FYVE domain-containing proteins and fill the international gap in the research on the functions of FYVE domain-containing proteins in oomycetes.

## 4. Materials and Methods

### 4.1. P. sojae and Plant Cultivation

The *P. sojae* isolates (P6497) used in this study were grown in 10% V8 juice agar at 25 °C in the dark. Soybean (Williams) provided by Brett Tyler (Department of Botany and Plant Pathology, Oregon State University, Corvallis, OR, USA) were grown in a glasshouse at 28 °C under a photoperiod of 16 h and 8 h of light and darkness, respectively.

### 4.2. Construction of Plasmids

The sgRNAs for CRISPR/Cas9-mediated gene knockout was designed on *EuPaGDT* (http://grna.ctegd.uga.edu, accessed on 20 June 2021) and then cloned into pYF515 plasmids following a previously described protocol [32]. The sequences 1 kb upstream and 1 kb downstream of the target gene were amplified and cloned into the pBluescript II KS^+^ donor vector and the replacement gene *NPTII* was inserted between them. The *PsFP1* overexpression complementation vector was constructed by amplification the full-length of *PsFP1* from cDNA from and then inserted into the PYF3 vector. The FYVE domain- or PX domain-truncated version of *PsFP1*, as well as the full-length version of *PsFP1,* were amplified from cDNA from and then inserted into the PYF3 vector fused with GFP for observation of subcellular localization. The sgRNAs and primers used in this study are listed in Tables S3 and S4.

### 4.3. P. sojae Transformation

The *P. sojae* transformation was conducted using previously described methods [32]. Briefly, transformants were generated using polyethylene glycol (PEG)-mediated protoplast transformation, and a V8 medium containing 50 μg ml^−1^ G418 was used to select positive transformants. For the gene knockout experiment, the G418-resistant transformants were cultured in a V8 liquid medium at 25 °C for 3 days, then the mycelia were collected for DNA or RNA extraction. Insertion of the gene into *P. sojae* was confirmed by PCR using the genomic DNA of transformants. For the subcellular localization experiment, the G418-resistant colonies were cultured in a V8 liquid medium for 2 days. The GFP signal images were captured under an Olympus FV3000 Spotlight Laser Scanning microscope with a 488 nm argon laser (emission wavelength 504–550 nm). At least three independent transformants were detected in each sample.

### 4.4. Phenotype Analysis of Transformants and P. sojae

Mycelial growth: A mycelial plug (5 mm in diameter) was deposited on a 10% V8 agar medium and the plates were incubated for 7 days at 25 °C in the dark. The diameter of the colony was then measured. Sensitivity to a series of temperatures and oxidative stress was tested by culturing P6497 and transformants on V8 plates in the dark at a range of different temperatures (4 °C, 12 °C, 20 °C, 25 °C, 28 °C, and 37 °C) or culturing them on V8 plates supplemented with 0, 2.5 or 5 mM H_2_O_2_. The inhibition rate = ([growth rate on plates without stress] − [growth rate on plates with stress])/growth rate on plates without stress [58].

Sporangium production: The P6497 and transformants were cultured on V8 plates at 25 °C in the dark for 7 days. The plates were then washed with distilled water seven times. Next, 10 mL of sterile water was added to the plates, and the isolates were incubated at 25 °C in the dark for 4 h. Sporangium production was determined by microscopy at 100× magnification.

Zoospore production: The P6497 and transformants were cultivated on 10% V8 plates for 10 days at 25 °C in the dark. The production of zoospores was induced by washing the plates with distilled water seven times, then adding 10 mL sterile water to the plates and incubating the isolates at 25 °C in the dark for 8 h. Zoospore production was quantified with a hemacytometer.

Cyst germination: The germination of the cystospores was determined by microscopy after overnight incubation on a water agar medium at 25 °C in the dark.

Oospore production: The P6497 and transformants were cultured on V8 plates in darkness at 25 °C for 7 days. The production of oospore was determined by microscopy at 100× magnification.

Pathogenicity: Virulence on soybean (Williams) leaves was determined by inoculating the first true leaves of 9- to 10-day-old soybeans with mycelial plugs (5 mm in diameter) from the edges of actively growing colonies or 10 μL of zoospore suspension (20 zoospores/µL). Virulence on etiolated soybean seedlings was determined by infecting etiolated soybean seedlings with 10 µL zoospore suspensions (20 zoospores/µL). Three days later, the size of the lesion was recorded and the area of the lesion was analyzed using ImageJ (National Institutes of Health, Bethesda, MD, USA).

All experiments were repeated at least three times, and the data were analyzed using a two-tailed Student’s *t*-test.

### 4.5. Quantitative Reverse Transcription Polymerase Chain Reaction Analysis

Materials in different stages including the mycelia (my), sporangia (sp), zoospores (zo), cysts (cy), germinated cysts (gc), and infection (IF 1.5, 3, 6, 12, 24, and 48 h) stages were collected as previously described [59]. Total RNA was extracted using an SV Total RNA Isolation kit (Promega, Madison, WI, USA) following the manufacturer’s instructions. The concentration and quality of the RNA samples were measured using a NanoDrop One microspectrophotometer (Thermo Fisher Scientific, Waltham, MA, USA). And the RNA integrity was also determined using agarose gel electrophoresis. Then 1µg of RNA was used as a template for first-strand cDNA synthesis using PrimeScriptTM Reverse Transcriptase (Takara, Shiga-Ken, Japan). Quantitative reverse transcription-polymerase chain reaction (qRT-PCR) was performed on a Bio-Rad CFX Connect Real-Time PCR System using TB Green Fast qPCR Mix (Takara, Shiga-Ken, Japan) with actin as a reference gene and the primers listed in Table S2. Data were analyzed using the 2^−ΔΔCT^ method [58]. Semi-qRT-PCR was performed on a Bio-Rad T100 using FastPfu DNA Polymerase (TransGen Biotech, Beijing, China) with actin as a reference gene.

### 4.6. Measurement of Hydrogen Peroxide Content in Mycelia

Intracellular H_2_O_2_ levels of the transformants and parental isolate were measured using an H_2_O_2_ content detection kit (Solarbio, Beijing, China). All isolates were inoculated with V8 juice liquid medium for 3 days and the mycelia were collected and washed twice in ddH_2_O. Next, a specified amount of the mycelia was fully ground in a mortar in an ice bath, after which reagents were added according to the instructions of the kit. The absorbance was then measured at 415 nm. All experiments were repeated at least three times.

### 4.7. Determination of Superoxide Dismutase (SOD) Activity and Catalase (CAT) Activity

The transformants and parental isolate were inoculated with V8 juice liquid medium for 3 days and the mycelia were collected and washed twice in ddH_2_O. Next, a specified amount of the mycelia was fully ground in a mortar in an ice bath, after which the homogenate was centrifuged at 4 °C for 10 min at 12,000× *g* and the supernatant was used to be protein concentration and enzymatic activity determined. The protein concentration was determined via Bradford Protein Assay Kit (Solarbio, Beijing, China). The SOD activity was determined by Total Superoxide Dismutase Assay Kit with NBT (Beyotime, Nantong, China), and the CAT activity was determined by Catalase Assay Kit (Beyotime, Nantong, China) according to the instructions of the kit. All experiments were repeated at least three times.

## 5. Conclusion and Perspective

In summary, we characterized the functions of FYVE domain-containing proteins PsFP1 and PsFP2 in *P. sojae* with CRISPR/Cas9 gene knockout system. Our results indicate that PsFP1 plays an important role in the vegetative growth, virulence, and oxidative stress response of *P. sojae*. Future studies are needed to further evaluate the roles and regulatory networks of PsFP1 during the vegetative growth, virulence, and oxidative stress response of *P. sojae* using bioinformatics, RNA-seq, and other approaches. Our results will enrich the understanding of the FYVE domain-containing proteins and fill the international gap in the research on the functions of FYVE domain-containing proteins in oomycetes.

## Data Availability

Not applicable.

## Supplementary Materials

The following are available online at https://www.mdpi.com/article/10.3390/ijms22126601/s1, Figure S1: The mycelial growth inhibition rate of the *PsFP1* knock-down mutants under different temperatures, Table S1: Characteristics of *PsFP1* knock-down transformants, Table S2: Characteristics of *PsFP2* knockout transformants, Table S3: sgRNA sequence used in the study, Table S4: Primers used in the study.
