# Supplementary material for: An FYVE-Domain-Containing Protein, PsFP1, Is Involved in Vegetative Growth, Oxidative Stress Response and Virulence of Phytophthora sojae
Source: Int J Mol Sci. 2021 Jun 20;22(12):6601. doi: 10.3390/ijms22126601 (PMC8233823; doi:10.3390/ijms22126601)
Supplement: Supplementary file 1 [file ijms-22-06601-s001.zip › ijms-1190145-supplementary/supplementary materials/Table S1-S4.pdf]

Table S1. Characteristics of *PsFP1* knock-down transformants.

Isolate	Mycelial growth (mm)	Sporangium production (no./field of view)	Zoospore production (10^4 /ml)	Cyst germination (%)	Oospore production (no./field of view)
P6497	45.1 ± 2.2a	11.4 ± 3.4 ab	3.7 ± 0.8 bc	77.0 ± 3.2abc	34.2 ± 4.7 ab
S1-7	31.3 ± 0.8b	8.9 ± 4.1 bc	10.5 ± 1.7 a	81.0 ± 5.5a	25.5 ± 5.6 d
S1-10	29.3 ± 2.1b	9.8 ± 4.4 ab	3.0 ± 0.5 bc	79.7 ± 2.6ab	32.0 ± 4.7 abc
S2-3	30.4 ± 0.7b	12.1 ± 4.6 ab	7.1 ± 1.5 a	72.6 ± 1.2bc	28.5 ± 4.3 bcd
S2-5-S6	32.4 ± 1.7b	11 ± 2.7 ab	4.5 ± 0.7 bc	81.1 ± 3.8a	26.5 ± 4.6 cd
S2-5-S51	30.8 ± 1.5b	13.9 ± 5.5 a	2.7 ± 0.5 bc	72.0 ±6.0c	30.5±7.1 abcd
S3-4	33.0 ± 1.8b	8.9 ± 3.4 bc	1.8 ± 0.3 c	81.4 ±4.8a	34.8 ± 3.5 a

Means in a column with the same letter indicate no significant different according to a Fisher's significant difference (LSD) test at P=0.05.

Table S2. Characteristics of *PsFP2* knockout transformants.

Isolate	Mycelial growth (mm)	Sporangium production (no./field of view)	Zoospore production (10^4 /ml)	Cyst germination (%)	Oospore production (no./field of view)
P6497	45.1 ± 2.2a	11.4 ± 3.4 ab	3.7 ± 0.8 bc	77.0 ± 3.2a	34.2 ± 4.7 cd
T4-48	44.8 ± 2.1a	9.4 ± 3.6 ab	3.4 ± 0.4 cd	78.5 ± 2.5a	45.3 ± 9.9 bcd
T4-49	46.1 ± 1.3a	12.9 ± 2.7 ab	4.7 ± 0.7 a	78.0 ± 2.0a	63.5 ± 9.1 ab
T5-8	36.3 ± 0.8b	19.3 ± 7.0 a	4.3 ± 0.3 ab	77.0 ± 3.1a	27.7 ± 2.9 d
T5-9	41.2 ± 1.4a	9.7 ± 1.8 ab	2.9 ± 0.3 d	77.6 ± 1.9a	68.0 ± 7.1 a
T5-13	46.2 ± 1.7a	10.7 ± 3.4 ab	3.5 ± 0.3 cd	78.5 ± 1.4a	59.7 ± 13.1 abc
T5-14	43.5 ± 2.7a	9.1 ± 3.2 bc	3.8 ± 0.5 bc	77.7 ± 3.1a	56.3 ±7.4 abc

Means in a column with the same letter indicate no significant different according to a Fisher's significant difference (LSD) test at P=0.05.

Table S3. sgRNA sequence used in the study

Primer	Sequence (5′-3′)	Application
PsFP1-SF1	CTAGCCCGCCCCTGATGAGTCCGTGAGGACGAAACGA GTAAGCTCGTCGGGCGGCGGGTGACGGATAC	sgRNA sequence for *PsFP1*
PsFP1-SR1	AAACGTATCCGTCACCCGCCGCCCGACGAGCTTACTCG TTTCGTCCTCACGGACTCATCAGGGGCGGG	
PsFP1-SF2	CTAGCGCCTTCCTGATGAGTCCGTGAGGACGAAACGA GTAAGCTCGTCGAAGGCCCAGATCCGTACCA	
PsFP1-SR2	AAACTGGTACGGATCTGGGCCTTCGACGAGCTTACTCG TTTCGTCCTCACGGACTCATCAGGAAGGCG	
PsFP1-SF3	CTAGCAACAAGCTGATGAGTCCGTGAGGACGAAACGA GTAAGCTCGTCCTTGTTGAACTCACCGATCA	
PsFP1-SR3	AAACTGATCGGTGAGTTCAACAAGGACGAGCTTACTC GTTTCGTCCTCACGGACTCATCAGCTTGTTG	
PsFP2-SF1	CTAGCTACAAACTGATGAGTCCGTGAGGACGAAACGA GTAAGCTCGTCTTTGTACAAGTAACTGGAAG	sgRNA sequence for *PsFP2*
PsFP2-SR1	AAACCTTCCAGTTACTTGTACAAAGACGAGCTTACTCG TTTCGTCCTCACGGACTCATCAGTTTGTAG	
PsFP2-SF2	CTAGCACCTTCCTGATGAGTCCGTGAGGACGAAACGA GTAAGCTCGTCGAAGGTCTTTATAACACTCC	
PsFP2-SR2	AAACGGAGTGTTATAAAGACCTTCGACGAGCTTACTCG TTTCGTCCTCACGGACTCATCAGGAAGGTG	
PsFP2-SF3	CTAGCGACATGCTGATGAGTCCGTGAGGACGAAACGA GTAAGCTCGTCCATGTCTCCCTCTGCAATTA	
PsFP2-SR3	AAACTAATTGCAGAGGGAGACATGGACGAGCTTACTC GTTTCGTCCTCACGGACTCATCAGCATGTCG	

Table S4. Primers used in the study

Primer	Sequence (5′-3′)	Application
PsFP1-qF	ATCAACTGGGACGAGGACACC	qRT-PCR analysis of *PsFP1*
PsFP1-qR	GTTCTGACGAGCCATCTTCTTCTG	
PsFP2-qF	TCGGATGGAGAGGATGATGACAAC	qRT-PCR analysis of *PsFP2*
PsFP2-qR	AGCAATGCGGACACTGAGACG	
PsAc-qF	ACTGCACCTTCCAGACCATC	qRT-PCR analysis of *PsActin*
PsAc-qR	CCACCACCTTGATCTTCATG	
PsFP1-DF1	GATAAGCTTGATATCGAATTCAGCATGGCTAAGCTCG	For *PsFP1* donor vector
PsFP1-DR1	GTTCAATCATCTAGACTCGGAGCTGCGCC	
PsFP1-DF2	CCGAGTCTAGATGATTGAACAAGATGGATTGCACG	
PsFP1-DR2	CTCTTTCTATTCAGAAGAACTCGTCAAGAAGGC	
PsFP1-DF3	GTTCTTCTGAATAGAAAGAGGACTGTAAGGAGATC	
PsFP1-DR3	CGCTCTAGAACTAGTGGATCCCCGAGAGCGATGTCCATC	
PsFP2-DF1	GATAAGCTTGATATCGAATTCCGGTCGAACCCATGT	For *PsFP2* donor vector
PsFP2-DR1	GTTCAATCATCAGCGATCCAGACGAGCG	
PsFP2-DF2	TGGATCGCTGATGATTGAACAAGATGGATTGCACG	
PsFP2-DR2	CTTACTCTCTTCAGAAGAACTCGTCAAGAAGGC	
PsFP2-DF3	GTTCTTCTGAAGAGAGTAAGTAGAAATTCACA	
PsFP2-DR3	CGCTCTAGAACTAGTGGATCCCGGCCCGCCTCGCGACGGAGAT	
PsFP1-F1	TGAGTAACACGGAGCCTATCG	Verify the *PsFP1* knock out transformants
PsFP1-R1	AGGAGCAAGGTGAGATGACAG	
PsFP1-F2	CATCAAGAGCGTTGTGAAGGAG	
PsFP1-R2	CCTGGTCAATGAAGCGAATGC	
PsFP1-F3	ATCGCCTTCTATCGCCTTCTTG	
PsFP1-R3	ACTTCCTTCTCCTCATTGATGCC	
PsFP2-F1	CGATCTTGGCAATCCACAGG	Verify the *PsFP2* knock out transformants
PsFP2-R1	CTTCAGCAATATCACGGGTAGC	
PsFP2-F2	CGGATGGAGAGGATGATGACAAC	
PsFP2-R2	ACTGGTAGGAGAAGGCAAATGG	
PsFP2-F3	GGATCTCCTGTCATCTCACCTTG	
PsFP2-R3	GCGTCACCAGAGCAGTCTTG	
PsFP1-GFP-F1	GATAGGCCTCCGCGGACTAGTATGGCAGGAAGTTCCGAGAAAGC	Insertion of *PsFP1* and *GFP* gene into PYF3 vector
PsFP1-GFP-R1	CCTTGCCCATGAACTCCTCCGAACGAGGCTTCG	
PsFP1-GFP-F2	GGAGGAGTTCATGGGCAAGGGCGAGGAA	
PsFP1-GFP-R2	AGAAGTAGGCACCGGTACCGGGCCCTCAACGCGTTCCGGAGTT	
C- *PsFP1*-F	GATAGGCCTCCGCGGACTAGTATGGCAGGAAGTTCCGAGAAAGC	Insertion of *PsFP1* into PYF3 vector
C- *PsFP1*-R	AGAAGTAGGCACCGGTACCGGGCCCCTAGAACTCCTC	

CGAACGAG
