# Supplementary figures and images for: An FYVE-Domain-Containing Protein, PsFP1, Is Involved in Vegetative Growth, Oxidative Stress Response and Virulence of Phytophthora sojae
Source: Int J Mol Sci. 2021 Jun 20;22(12):6601. doi: 10.3390/ijms22126601 (PMC8233823; doi:10.3390/ijms22126601)

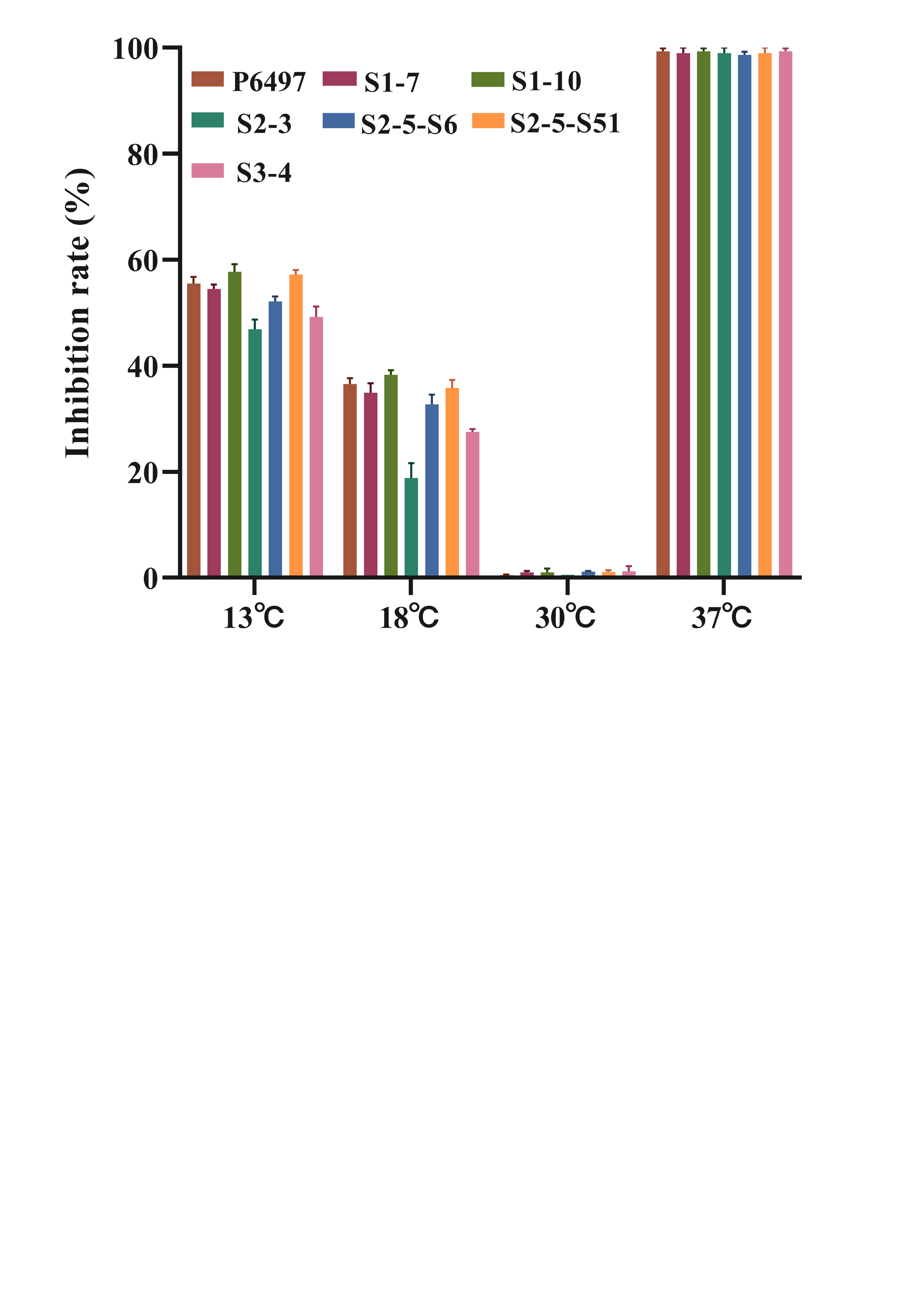

Supplement: Supplementary file 1 [file ijms-22-06601-s001.zip › ijms-1190145-supplementary/supplementary materials/Fig S1 The mycelia growth inhibition rate of the PsFP1 knock-down mutants under different temperature.tif]
